# Nutritional Knowledge and Attitudes of Adolescent Swimmers in Trinidad and Tobago

**DOI:** 10.1155/2014/506434

**Published:** 2014-02-11

**Authors:** Marquitta C. Webb, Safiya E. Beckford

**Affiliations:** Department of Agricultural Economics and Extension, Faculty of Food and Agriculture, The University of the West Indies, St. Augustine, Trinidad and Tobago

## Abstract

*Purpose*. To investigate the level of nutrition knowledge and attitude of adolescent male and female swimmers training competitively in Trinidad and Tobago. *Methodology*. A self-administered questionnaire, which consisted of 21 nutrition knowledge and 11 attitude statements, was utilized to assess the level of nutrition knowledge and attitude of adolescent swimmers. For the assessment of nutrition knowledge, correct answers were given a score of “1” and incorrect answers were given a score of “0.” For the evaluation of attitude towards nutrition, a score ranging from 1 to 5 was assigned to each response; “5” was given to the most positive response, and “1” was given to the most negative. Data were analyzed using SPSS version 21.0. *Results*. Two hundred and twenty swimmers with a mean age of 14.56 ± 2.544 completed the questionnaire. The mean nutrition knowledge score was 10.97 ± 2.897 and mean attitude score was 41.69 ± 6.215. Nutrition knowledge was positively and significantly related to the number of reported nutrition sources (*r* = 0.172, *P* = 0.005). Nutrition knowledge was positively and significantly related to the attitude (*r* = 0.130, *P* = 0.027). *Conclusions and Implication*. Athletes lack nutrition knowledge but have a positive attitude towards nutrition, which may indicate receptiveness to future nutrition education.

## 1. Introduction

Adolescence is the stage of life between ages of 11 and 21 years [1]. During this time, significant changes occur, which prepares a child for adulthood. The rapid growth and development during this stage increases energy and nutrient requirements [2–4]. Additionally, when an adolescent is an athlete, there are several important factors to consider in relation to nutrition for both growth and sports performance. Factors, such as lifestyle and dietary habits, have a tremendous impact on health status and sports performance [3]. As such, to sustain proper growth, development, and health while improving performance, adolescents engaged in sports could benefit from knowledge about the importance of good nutrition.

Nutrition is an important factor in the performance and health of athletes. High levels of nutrition knowledge and positive attitudes can result in increased performance and health of an athlete. Therefore, nutrition is an essential component of any athletic or physical activity program. Almost every process in the body involves some aspect of nutrition. Hence, knowledge of how the body utilizes nutrients and the relation of nutrients with metabolism in health and disease, energy production, and recovery from exercise are all important aspects of sports nutrition.

Existing studies [5, 6] are constant in their findings that athletes lack the nutrition knowledge they need in order to gain the benefits of increased performance and health. This may be due to misconceptions about nutrition and its importance to health and performance among athletes [7–13]. Additionally, it may be due to deficiencies in nutrition knowledge and misguided or incorrect information given by one or more unreliable sources [7–13]. Torres-McGehee and colleagues [14] examined nutrition knowledge in 4 domains of sports nutrition among athletes in various sports as well as coaches, athletic trainers, and conditioning specialists and found that athletes had inadequate nutrition knowledge scores of less than 75%. Hoogenboom and colleagues [15] investigated the nutritional knowledge and eating behaviors of female collegiate swimmers in Divisions I, II, and III and reported that on average these swimmers had a mean nutrition knowledge score of 54.53 ± 4.34, which was considered fair. These researchers concluded that these swimmers exhibited a lack of application of the knowledge they possessed. Areas in which adolescents typically lack nutrition knowledge include the dietary roles of protein and carbohydrates, role of vitamin and minerals, carbohydrate loading, glycemic index, muscle energy sources, energy expenditure and nutritional requirements, nutrient sources, and nutrients and performance [5, 8, 16]. Ozdoğan and Ozcelik [6] evaluated the nutrition knowledge of students in the sports teaching and coaching department at three universities using 30 true/false questions and found that the mean nutrition knowledge score was low (12.247 ± 3.525) and showed insufficiency of knowledge. Similarly, in a study of 185 Mid-American Conference College female softball players, which measured nutrition knowledge, practices, attitudes, and information sources using a questionnaire, Torres-McGehee and colleagues [14] found that the mean nutrition knowledge score was low (45.7 ± 4.7). However, when a standard 60% cut-off-point was set as the failing mark, two thirds (*n* = 120, 62%) of the softball players were judged as having failed the questionnaire, indicating a low level of nutrition knowledge. Likewise, Davar [16] reported that the total mean score for female hockey players was 22.85 (38.8%) and concluded that athletes lack nutrition knowledge. In contrast, Azizi and colleagues [9] utilized a questionnaire to investigate nutritional knowledge and attitudes of elite college athletes and reported that males and females had moderate nutrition knowledge, 52.36 ± 6.7 and 54.31 ± 6.3, respectively. However, although the scores were classified as moderate, the researchers concluded that nutrition knowledge needs to be improved in this population. In another study, Zawila and colleagues [8] indicated that female collegiate cross-country runners had a reasonable amount of knowledge regarding some aspects of nutrition. However, there were parts in which the lack of knowledge was evident. Runners scored higher in sections related to sport nutrition than sections related to general nutrition. Additionally, those exposed to nutrition courses in college showed a greater score for nutrition knowledge than those who were not exposed to nutrition class.

Besides knowledge, attitude towards nutrition is also an important factor in the performance of an athlete. There is a growing body of evidence that suggests athletes hold a positive attitude towards nutrition [12, 17, 18]. Azizi and colleagues [9] noted that nutrition attitude scores for males and females were 50.61 ± 5.1 and 52.03 ± 5.8, respectively, which demonstrated positive attitudes toward nutrition. Females were found to have a significant higher attitude score than males, which corresponded to a better attitude towards nutrition. Zawila and colleagues [8] conveyed that female collegiate cross-country runners had a mean total positive response for attitudes of 90.6% representing an overall positive attitude towards nutrition. The mean attitude towards nutrition score in the investigation of college softball players by Hornstrom and colleagues [11] was 1.9 ± 0.4 indicating a positive attitude (range 1–6, 1 signifying a more positive attitude and 6 signifying a more negative attitude). Davar [16] reported that the mean of female hockey players' total positive attitude response was 90.6% and 93.3% of the players agreed that providing nutrition education would have a positive impact on food selection. Thus, several authors have shown that athletes have a positive attitude towards nutrition and therefore may be open to acquiring nutrition information [8, 16].

There is a relationship between nutrition knowledge and attitude. This statement was supported by studies [9, 11] which indicated that nutrition knowledge and attitudes are significant and positively correlated; that is, the more the knowledge about nutrition the greater the positive attitude towards it. However, nutrition knowledge and attitudes had no impact on dietary practices [11].

A review of literature suggests that few studies have been conducted to explore the nutrition knowledge and attitude towards nutrition of athletes in developing countries, such as Trinidad and Tobago. Most of the literature focuses on elite and college level athletes with few articles on their adolescent counterparts. Additionally, there is no current information about the level of nutrition knowledge among adolescent swimmers in Trinidad and Tobago. Hence, the purpose of this study was to assess the level of nutrition knowledge and attitude of adolescent swimmers training competitively in Trinidad and Tobago. Study aims were to enable participants to examine their nutrition knowledge and attitude towards nutrition in a quest to enhance performance. Study hypotheses were that (1) adolescent competitive swimmers will lack nutrition knowledge, (2) adolescent competitive swimmers will have a positive attitude toward nutrition, and (3) there will be a positive correlation between nutrition knowledge and attitude toward nutrition.

## 2. Materials and Methods

### 2.1. Subjects

The questionnaire to evaluate the level of the nutrition knowledge and attitudes and nutrition of adolescent swimmers was carried out from September 2012 to November 2012. The participants involved were registered and trained competitively with private swimming clubs in Trinidad and Tobago. All of the clubs were registered with the Amateur Swimming Association of Trinidad and Tobago (ASATT). A total of 220 adolescent male and female swimmers, aged 11–21 years, were recruited for this study. To guarantee anonymity of responses and easy identification of the questionnaires by participants, identification numbers were randomly assigned to each questionnaire. The same instructions were read to each participant to ensure the questionnaire was completed correctly. Each questionnaire took approximately 15 minutes to complete. Ethical approval was obtained from the ethics committee. Additionally, signed informed consent was obtained from participants and/or their parent/guardian.

### 2.2. Procedure

In this cross-sectional descriptive study, a self-administered structured questionnaire was utilized to evaluate the nutrition knowledge and attitudes of adolescent swimmers training in competitive clubs. The survey instrument used in this study was developed from a combination of previously administered questionnaires [6, 8]. The modified questionnaire had a reliability of 0.71 and a review by faculty members was utilized to establish its face validity. The questionnaire items included demographic characteristics of participants, 21 nutrition knowledge statements, to which participants replied “true,” “false,” or “not sure,” and 11 statements to evaluate the attitudes toward nutrition, to which participants responded using a Likert scale with options ranging from “strongly agree” to “strongly disagree.” For the assessment of nutrition knowledge, correct answers were given a score of “1” and incorrect answers were given a score of “0.” For the evaluation of attitude towards nutrition a score ranging from 1 to 5 was assigned to each response; “5” was given to the most positive response and “1” was given to the most negative.

### 2.3. Statistical Analysis

All data were analyzed using the Statistical Package for Social Sciences (SPSS version 21.0, SPSS Inc., Chicago, IL, 2012). Descriptive statistics were used to compile the data collected. Analysis of variance (ANOVA) (confidence interval 95%) was used to compare nutrition knowledge and attitudes between sex, age range, highest level of education, and number of nutrition classes attended. Pearson correlation was used to evaluate the relationship between nutrition knowledge and sources of information and between nutrition knowledge and attitudes.

## 3. Results

### 3.1. Demographic Profile

Table 1 presents the demographics of the study population. Of the 220 swimmers who participated in the study, 122 (55.5%) were males and 98 (44.5%) females with a mean age of 14.56 ± 2.544. The majority (58.6%) of the participants were of mixed descent. Most of the respondents attended secondary school and were in the “15–17” age group. Over half (56.8%) of the participants had never had a nutrition class/course/seminar since beginning to swim. Additionally, more than half (53.2%) of the participants indicated never attending a nutrition class/course/seminar over the last year.

### 3.2. Nutrition Knowledge

The highest potential score for the nutrition knowledge was 21 and the lowest score was 0. The mean nutrition knowledge score for the entire sample was 10.97 ± 2.90 (Table 2). The highest score was 20 (95.24%) and the lowest was 2 (9.52%). Male participants had a mean score of 11.05 ± 2.793, which was higher than the female participants' mean score of 10.88 ± 3.033. Participants in the age group of 18–21 scored the highest out of the 4 specified age groups with a mean score of 12.97 ± 2.735. The 11-12 age group scored the lowest with 9.95 ± 2.362.  Table 3 presents survey questions that were answered correctly less than 50% by the respondents. Participants scored below 50% for 10 of the 21 nutrition knowledge questions. There were no significant differences in the knowledge scores when compared between sex, age range, and highest level of education, however, there was a significant difference in knowledge scores between the categories (i.e., none, 1–3, 4 or more) for of number of nutrition classes attended. Hence, the more nutrition classes attended, the higher the nutrition knowledge scores. Pearson correlation revealed that nutrition knowledge was positively and significantly related to the number of reported nutrition sources (*r* = 0.172, *P* = 0.005). Additionally, nutrition knowledge was positively and significantly related to the attitude of the respondents held towards nutrition (*r* = 0.130, *P* = 0.027).

### 3.3. Attitude towards Nutrition

The highest possible score for the attitude section was 55 and the lowest was 11. The mean positive attitude towards nutrition score, standard deviation, and ANOVA results are shown in Table 4 according to sex and age groups. The mean score for attitude towards nutrition for the entire sample was 41.69 ± 6.215. The highest score was 55 (100%) and the lowest 19 (34.55%). Male respondents had a slightly higher mean positive attitude towards nutrition score than female respondents, 41.96 ± 5.931 and 41.35 ± 6.566, respectively. As the age range increased so did the attitude towards nutrition scores. The age range of “18–21” had the highest score of 43.83 ± 6.438, followed by the “15–17” age range scoring 42.47 ± 6.236. The “11-12” and “13-14” age ranges had similar scores of 40.48 ± 6.239 and 40.47 ± 5.618, respectively. There were no significant differences in the attitude towards nutrition score between the means for sex, age range, highest level of education, number of nutrition classes attended since beginning to swim, and club.

## 4. Discussion

This current study investigated the nutrition knowledge and attitudes of adolescent swimmers training competitively in Trinidad and Tobago. Out of the 21 nutrition knowledge statements set, there were 5 statements that dealt specifically with macronutrients, 8 with micronutrients, and 2 with hydration, while the remaining 6 statements focused on diet practices. From the analysis of results, the nutrition knowledge of adolescent swimmers in this study was found to be insufficient. The mean knowledge score for the general sample was 10.97 ± 2.897, which was lower than the mean score of 12.247 ± 3.525 found in Ozdoğan and Ozcelik [6] for the same knowledge statements. In another study [5], the mean score for male respondents was higher than that for female participants. However, after comparison of means, there were no significant differences between genders. The mean knowledge score increased as the age range increased, but no significant difference was found between means. The mean scores for participants with or without nutrition classes were also compared (means not shown in results) and were found to be statistically significant (*P* = 0.039). This indicated that the more nutrition classes taken, the higher the score, which suggested that nutrition education may be beneficial to these groups of athletes. The areas of nutrition knowledge that seemed to be deficient in this sample, as demonstrated by less than 50% of participants responding correctly, were the role of protein in the body, the characteristics of carbohydrates, the role of micronutrients in the body (especially iron), types of fats, and proper energy sources from food.

Carbohydrates, in the form of glucose and glycogen, are the main sources of energy for muscle; proteins are only used for energy when the body is deficient in carbohydrates [8], which is important for athletes to know. For the statement “protein is the main energy source for the muscle,” only 20% of the respondents answered correctly. This was similar to the 31.7% of female cross-country runners who correctly answered this statement [8]. In contrast, Ozdoğan and Ozcelik [6] found that 77.8% of the athletes answered this statement correctly. However, a diet rich in carbohydrates increases endurance performance because of the extra store of glycogen in the muscles. For the statement “carbohydrates are stored in muscles in the form of glycogen,” 46.4% of participants answered correctly. The amount of carbohydrates consumed affects the amount of glycogen stored; if glycogen stores are low, performance can be negatively affected [19].

Although participants were fairly knowledgeable about the importance of fat in the diet they were not very well familiar with the types of fat, which was also reported by Davar [16] and Dunn and colleagues [20]. It is recommended that 30% of an athlete's caloric intake be from fat sources [21]. Fats have many roles in the body; however, saturated fats have been linked to heart disease due to their potential role in increasing LDL cholesterol. Hence, it is recommended that saturated fats be replaced with unsaturated fats (mono- and polyunsaturated fats) in the diet, which decreases the risk of heart disease [19]. For the statement “saturated and unsaturated oils both have an equal effect on the health,” only 31.4% of the participants answered correctly.

Only 30% of the swimmers chose “false” for the statement, “table salt is an essential part of a healthy diet,” which was similar to the 37.6% in Ozdoğan and Ozcelik [6]. Sodium is the major extracellular cation and is needed for nerve impulses and muscle contractions, maintenance of cellular volume, and acid base balance [19]. Sodium is naturally found in vegetables and cereals and consuming these items can provide the recommended amount of sodium making it unnecessary to have additional salt added to food. Additionally, salt (sodium chloride) has been linked to high blood pressure and edema, so excess amounts should be avoided [6].

The statement “iron-deficiency anemia results in a decrease in the amount of oxygen that can be carried in the blood” was not answered well by the participants with only 38.2% correctly choosing “true.” In contrast, a similar question, “iron deficiency anemia results in decreased activity and performance,” was answered well by participants in Davar's study [16]. Less than 40% of swimmers (37.7%) correctly chose “false” for the statement “iron in meat is absorbed at the same rate as iron in a plant food.” This showed a lack of knowledge about sources of iron, which is supported by the results in Davar's study [16]. Iron is needed for creation of hemoglobin and myoglobin, which accepts transports and releases oxygen around the body; it also serves as a cofactor for many enzymes needed for metabolism [19]. Absorption of iron is partially dependent on its source; animal sources of iron contain heme, which is easily absorbed, and plant sources contain nonheme iron, which is not absorbed as well (animal sources also contain nonheme iron) [19]. A deficiency in iron is one of the most common nutrient deficiencies and is observed in many athletes and nonathletes, especially in females [6].

Only 10.5% of the participants correctly chose “false” for the statement “vitamin supplements are recommended for all physically active people,” which was the case in Zawila and colleagues [8] where only 10.0% of the participants correctly responded to the statement. In contrast, 67.9% of the participants in the study by Ozdoğan and Ozcelik [6] correctly answered the same question. For the statement “vitamins are good sources of energy” only 13.2% of the participants correctly identified “false” was the answer. This was lower than the 31.6% and 64.1% found in other studies [6, 8]. Nutrient supplementation does not benefit the performance of well-nourished athletes, so it can be said that if a balanced diet is achieved which meets caloric needs of the athlete, additional vitamin supplements are not required [19]. Vitamins also do not produce energy; however, they are needed in the processes required for energy production [20].

Swimmers who correctly selected “false” to the statement “during physical activity, feeling thirsty is enough to indicate the need for liquid” were a mere 20.5% of the sample. In the study performed by Ozdoğan and Ozcelik [6], the rate of people being knowledgeable about this was more than half the rate seen in the present study. However 85.9% of swimmers correctly indicated “true” as the response to “dehydration decreases performance,” which was also the case in the Davar study [16] where it was reported that female hockey players were seemingly knowledgeable about this. In normal circumstances, thirst may be enough to stimulate fluid intake; however, in vigorous training situations and demanding environments thirst may be an inadequate indicator [22]. When an athlete is well hydrated before, during, and after exercise performance is optimal. However, if dehydrated performance decreases, there is an increased risk of potentially life-threatening heat related injury [23]. The swimmers in this study need to be educated about the importance of staying hydrated before, during, and after exercise.

The statement “basic sugars like brown or granulated sugar, jam, and honey are the most suitable energy sources for sportsmen” was correctly answered by 43.2% of the participants which was lower than the 72.3% of athletes that correctly answered in Ozdoğan and Ozcelik [6]. Foods like basic sugars, jam, and honey are high glycemic index foods, which enter the blood stream quickly and are carried away by insulin to the muscles; they cannot be used to maintain blood glucose levels over a long period of time and may cause fatigue [24]. Athletes should consume foods of moderate to low glycemic index, which are broken down over time and are able to provide a constant supply of glucose in the blood over a period of time [24]. Athletes in this study should also be educated about pre-, during, and postexercise nutritional practices, including foods with better glycemic indices.

In the results for the attitude section there were overall positive responses for 7 out of the 11 questions. The mean score of 41.69 ± 6.215 reflected an overall positive attitude towards nutrition. There was no significant difference found between any of the means when compared between sex, age groups, level of education, and previous exposure to nutrition classes. However, in another study it was reported that female athletes had a significantly higher attitude score than male athletes and also those enrolled in physical activity majors had a higher score than other majors [9].

For the statement “learning facts about nutrition is the best way to achieve favorable changes in food habits” 79.5% of participants either strongly agreed or agreed with the statement which was slightly less than in Davar [16] where 91.7% and 93.3% of athletes strongly agreed or agreed, respectively. Similarly, Zawila and colleagues [8] reported higher results for the same statement. There was a low acceptance (16.3%) of the statement “learning about nutrition is not important for athletes because they eat so much food and they always get the nutrients their bodies need” and a high acceptance of the statement “the relationship between good eating habits and good health should be stressed to the athlete” by the swimmers. The results to these statements showed that the athletes may be receptive to nutrition education. The same conclusion was made by Davar [16] and Zawila and colleagues [8] who found that there was a significantly positive correlation between nutrition knowledge and attitudes (*P* = 0.027). Many studies [9, 11, 25] have found the same relationship. Therefore, it may be said that nutrition education may not only increase nutrition knowledge but also have a positive effect on attitudes.

The current study is not without limitations. Studies have shown no relationship between nutrition knowledge and food behavior but a few do [26, 27]. However, in this study, we did not assess the relationship between knowledge, attitudes, and behaviors, or, more basically, food choices. There are several factors that influence food selection and nutrition, including flavor, religion, culture, and cognitive and social factors. Therefore, knowledge does not always translate into good food choices since athletes with a wealth of nutrition knowledge still eat poorly because of other factors. Another limitation is that we did not study the application of attitudes. There is no assumption that if knowledge is increased and attitude is good, the athlete will go on and make excellent food choices.

## 5. Conclusion

The findings serve as an indication of the need for nutrition education among swimmers and possibly other athletes. Athletes face enormous pressure to perform at their optimal level. However, in order to reach that optimal performance level the athletes must be equipped with excellent support which may be influenced by knowledge and attitude. Swimmers in Trinidad and Tobago were found to have inadequate nutrition knowledge, which could potentially contribute to their improved performance and health. Nutrition plays a critical role in the performance of an athlete; therefore by improving their knowledge about nutrition and good food choices, athletes could enhance their chances of reaching optimal level. Based on the poor nutrition knowledge but positive attitude towards nutrition, the swimmers may benefit from a nutrition education intervention program, which is geared towards improving eating habits for optimal performance and healthy lifestyle changes.

## 6. Implications for Research and Practice

Findings suggest that nutrition education should be a component of an athlete's training program. Data from this study provided some useful information which can be used to develop areas of an intervention program for swimmers. Future studies need to determine whether athletes in other sports possess the nutrition knowledge and attitudes toward nutrition that will enable them to perform at their optimal level.

## Figures and Tables

**Table 1:** Profile of the study population (*n* = 220).

Variable	Classification	Frequency (*n* = 220)	Percentage (%)
Sex	Female	98	44.5
	Male	122	55.5

Ethnicity	African	70	31.8
	Indian	11	5.0
	Caucasian	7	3.2
	Asian	3	1.4
	Mixed	129	58.6

Age range	11-12	61	27.7
	13-14	59	26.8
	15–17	70	31.8
	18–21	30	13.6

Highest level of education	Primary	16	7.3
	Secondary	175	79.5
	Tertiary	29	13.2

Number of nutrition classes/courses/seminars attended	None	125	56.8
	1–3	76	35.0
	4 or more	18	8.2

Most recent class/course/seminar attended	Never	117	53.2
	2011-2012	74	33.6
	More than 2 years ago	29	13.2

**Table 2:** Mean (SD) nutrition knowledge score according to the variables (*n* = 220).

Category	Group	Means ± standard deviation	P**
Sample	Total	10.97 ± 2.897	

Sex	Male	11.05 ± 2.793	0.880
	Female	10.88 ± 3.033	

Age range	11-12	9.95 ± 2.362	0.158
	13-14	10.78 ± 3.074	
	15–17	11.17 ± 2.823	
	18–21	12.97 ± 2.735	

**P < 0.05.

**Table 3:** Questions answered correctly by less than 50% of the participants (*n* = 220).

Statement	Frequency (*n* = 220)	Mean ± standard deviation
Protein is the main energy source for the muscle.	44 (20.0%)	0.20 ± 0.401
Iron-deficiency anemia results in a decrease in the amount of oxygen that can be carried in the blood.	84 (38.2%)	0.38 ± 0.487
Iron in meat is absorbed at the same rate as iron in a plant food.	83 (37.7%)	0.38 ± 0.486
Vitamin supplements are recommended for all physically active people.	23 (10.5%)	0.10 ± 0.307
During physical activity, feeling thirsty is enough to indicate the need for liquid.	45 (20.5%)	0.20 ± 0.404
Vitamins are good sources of energy.	29 (13.2%)	0.13 ± 0.339
Saturated and unsaturated oils both have an equal effect on the health.	69 (31.4%)	0.31 ± 0.465
Table salt is an essential part of a healthy diet.	66 (30.0%)	0.30 ± 0.495
Basic sugars like brown or granulated sugar, jam, and honey are the most suitable energy sources for sportsmen.	95 (43.2%)	0.43 ± 0.496
Carbohydrates are stored in muscles in the form of glycogen.	102 (46.4%)	0.46 ± 0.500

**Table 4:** Mean (SD) positive attitude towards nutrition scores according to the variables (*n* = 220).

Category	Group	Means ± standard deviation	P**
Sample	Total	41.69 ± 6.215	

Sex	Male	41.96 ± 5.931	0.707
	Female	41.35 ± 6.566	

Age range	11-12	40.48 ± 6.239	0.074
	13-14	40.47 ± 5.618	
	15–17	42.47 ± 6.236	
	18–21	43.83 ± 6.438	

**P < 0.05.
